# Visual analysis of mesenchymal stem cell research in liver disease based on bibliometrics

**DOI:** 10.1016/j.iliver.2022.11.006

**Published:** 2022-11-23

**Authors:** Chengzuo Han, Rui Wang, Nan Xu, Xuyong Wei, Qiang Wei, Xiao Xu

**Affiliations:** a Key Laboratory of Integrated Oncology and Intelligent Medicine of Zhejiang Province, Department of Hepatobiliary and Pancreatic Surgery, Affiliated Hangzhou First People's Hospital, Zhejiang University School of Medicine, Hangzhou, 310006, China; b Westlake Laboratory of Life Sciences and Biomedicine, Hangzhou, 310024, China

**Keywords:** Mesenchymal stem cells, Liver disease, Bibliometrics, Visualization

## Abstract

The research of mesenchymal stem cells (MSCs) in the field of liver diseases has received more and more attention. This paper introduces the current situation, hot spots, and development trends in this field. Comprehensive searches were conducted using Web of Science Core Collection from January 1, 2000 to December 13, 2021 with the following keywords: TS(topic) = (liver∗OR hepatic∗OR hepatocyte) AND TS(topic) = (Mesenchymal stem cell∗). VOSviewer (version 1.6.16) and CiteSpace V are used as bibliometric tools to analyze and visualize the knowledge graph. A total of 4452 papers were included in this study, and the number of research papers on MSCs in the field of liver diseases increased from January 2000 to December 2020. Eighty-four countries and regions have published articles on research in this field, among which China and the United States are the main two countries of publication. Based on the keyword burst detection, we find that the research in this field has shifted from basic research to clinical application, from medical research to interdisciplinary research. Tissue engineering and regenerative medicine are the frontier fields of MSCs research in liver diseases. Multicountry, multi-author cooperation, and multi-disciplinary intersection are the research trends in this field. Exocrine body, obesity, and tissue engineering are the hotspots in this field.

## 1 Introduction

Mesenchymal stem cells (MSCs), a class of adult stem cells with multidirectional differentiation and self-replication ability, are a promising strategy for the treatment of liver diseases according to recent research. Studies about MSCs started in 1916 [1] and were first reported in the context of human cirrhosis in the journal *Nature* in 1962 [2]. In the past two decades, 78,680 articles relating to MSCs were published, of which 4452 (5.66%) articles were reported in the field of liver disease across all journals.

MSCs are abundant and can be found in almost all organs and tissues, such as umbilical cord, fat, and bone marrow (BM) [3]. MSCs have low immunogenicity and mainly inhibit the proliferation, differentiation, and function of adaptive immune cells [4,5]. Accordingly, MSCs are capable of monitoring and regulating inflammatory responses [6]. Recent studies showed that the tissue repair and immunomodulatory functions of MSCs provide a promising strategy for the treatment of liver diseases. MSCs can inhibit chronic liver inflammation and fibrosis by regulating the proliferation and apoptosis of hepatic stellate cells (HSCs), transforming growth factor β secretion, and collagen deposition [7]. MSCs treat non-alcoholic fatty liver by improving insulin resistance, regulating lipid metabolism, regulating glucose metabolism, and inhibiting fibrosis through paracrine mechanisms [[8], [9], [10], [11]]. MSC-derived exosomes have the advantages of being smaller, less complex, and easier to produce and store cells and, therefore, may serve as an ideal treatment for liver diseases. Exosomes derived from human umbilical cord MSCs have the capacity to inhibit HSCs from becoming activated by the transforming growth factor β/Smad signaling pathway, which inhibits the proliferation of HSCs in injured liver, lowers collagen formation, and slows the progression of hepatic fibrosis [12].

Bibliometrics studies the quantitative relationship, distribution structure, and change law of a literature collection using statistical analysis, network analysis, and graph theory methods on the document system as the research object. It describes the internal structure of scientific literature and uses quantitative indicators to reflect its quantitative characteristics and laws, allowing us to understand the research level of scientific research subjects, distribution of scientific research achievements, and research hotspots within an entire scientific field. With the recent development and updating of visualization tools, bibliometrics has become widely used in the field of biomedicine. Indeed, it is extremely useful for studying disease mechanisms and developing clinical guidelines. Recently, research on literature visualization about MSCs has also emerged. Zhang X et al. focused on MSC-derived extracellular vesicles and proposed that future research should be focused on aging [13]. Chen C et al. used visualization tools to examine MSC research in cardiovascular disease and discovered that engineering and materials disciplines are current research hotspots [14]. MSCs were also identified by Shao B. et al. as one of the most important drugs for future liver disease treatments in the first bibliometric research in the field of liver disease [15]. Therefore, it is necessary to use visualization tools to analyze the field from multiple perspectives. CiteSpace is a knowledge graph visualization tool developed by Dr. Chen Chaomei using the Java platform [16], while VOSviewer(VOSviewer - Visualizing scientific landscapes) [17] is a knowledge graph drawing tool developed by Van Eck and Waltman from the Netherlands. Using these two tools and bibliometrics, we analyzed trends and hotspots of global publications on MSCs in the field of liver diseases from January 1, 2000 to December 13, 2021.

## 2 Method

This bibliographic review included original articles and reviews on MSCs in liver disease. We obtained a record of 4452 manuscripts published between January 1, 2000 and December 13, 2021 from the Web of Science Core Collection. Searches were conducted using the following keywords: TS (topic)= (liver∗ OR hepatic∗ OR hepatocyte) AND TS (topic)= (Mesenchymal stem cell∗). In this paper, VOSviewer (version 1.6.16) and CiteSpace were used as bibliometric tools to analyze the visual knowledge graph of the documents retrieved from the literature database. Both CiteSpace and VOSviewer are visualization tools that can be used to draw knowledge graphs [13,14]. VOSviewer is easy to operate and can construct key information co-occurrence networks and density analysis for the selected documents. CiteSpace can analyze literature co-citation by processing time slices to show the developmental trends of key information. Therefore, by combining these two tools to visually analyze the selected literature, we can more clearly grasp the research progress and development trends of MSCs in the field of liver disease.

## 3 Results

### 3.1 Year of publication

**Fig. 1:**
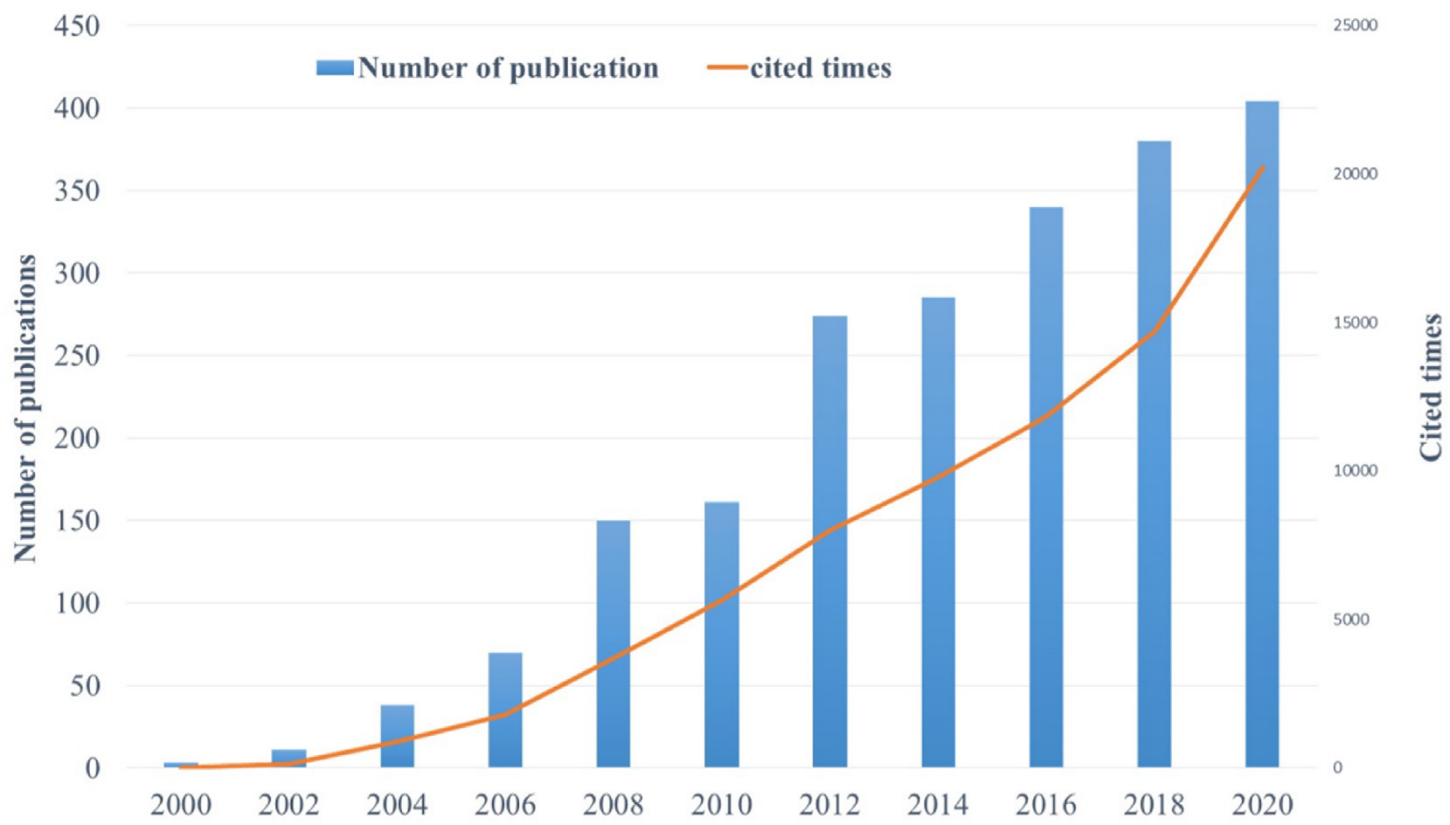
Trends for numbers of academic papers and citations on mesenchymal stem cells in liver disease from 2000 to 2020.

A total of 4452 papers were included in this study and the number of research papers on MSCs in the field of liver disease increased from January 2000 to December 2021 (Fig. 1). Because they are affected by the retrieval time, statistics for the number of articles published in 2021 are incomplete. The first paper describing research in this field was published in 2000. As shown in Fig. 1, the frequency of literature citations published between January 2000 and December 2020 also increased. On the whole, research on MSCs in the field of liver disease continues to increase, indicating that scholars pay high attention to this field. The number of publications climbed by over 100 every five years since 2004, and the numbers of times these publications were cited sharply increased (by over 30%) from 2018 to 2020, reflecting positive trends of development in the field, especially in the most recent 5 years.

### 3.2 Country and region

Research groups from 84 countries and regions published 4452 articles on MSCs in liver disease. The top 10 countries, consisting of four Asian countries, four European countries, and two North American countries, published a total of 4207 papers, accounting for 94.50% of the total papers published. The top three countries are China, the United States, and Japan, which published 1490, 858 and 381 articles, respectively, accounting for 33.468%, 19.272%, and 8.558% of the total number of articles, respectively.

### 3.3 Authors and institutions

**Fig. 2:**
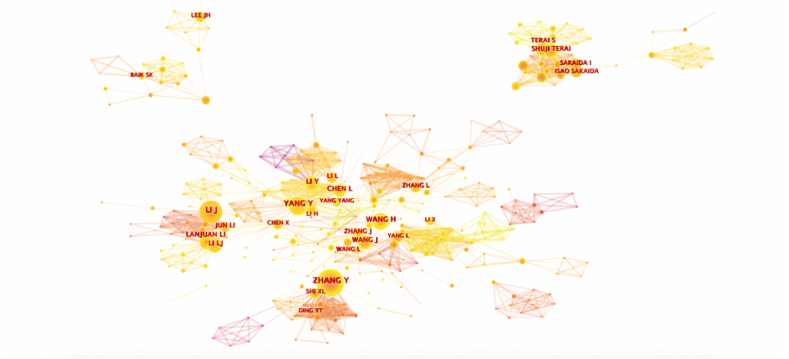
Author cooperation network in studies of mesenchymal stem cells in liver disease.

Author co-occurrence analysis can identify the cooperation and cross-citation relationship between core figures and researchers in a research field. In the author cooperation network, the node size represents the number of articles, while the line thickness represents the cooperation frequency between authors (i.e., the thicker the line, the higher the cooperation frequency). In the network, N represents the number of nodes, while E represents the number of lines. Higher line densities indicate a closer cooperative relationship between authors. Fig. 2 shows the co-occurrence network detection results of authors, with N = 1045 and E = 3629 (density = 0.0067), representing 902 significant study authors. There are 3629 lines in the network, most of which are closely connected, forming several representative core author groups. As highlighted in Fig. 2, the scholars who have published more than 10 articles include Lanjuan Li, Jun Li, Xiaolei Shi, Hossein Baharvand, Shuji Terai, Hongcui Cao, Mustapha Najimi, and others, of which members of Li Lanjuan's team (34) have published the largest number of articles. Influential scholars include Li Li, Jin Yang Gu, Daniel R. Meldrum, Lanjuan Li, and others producing a citation burst in the map.

### 3.4 Co-citation and cluster analysis

**Fig. 3:**
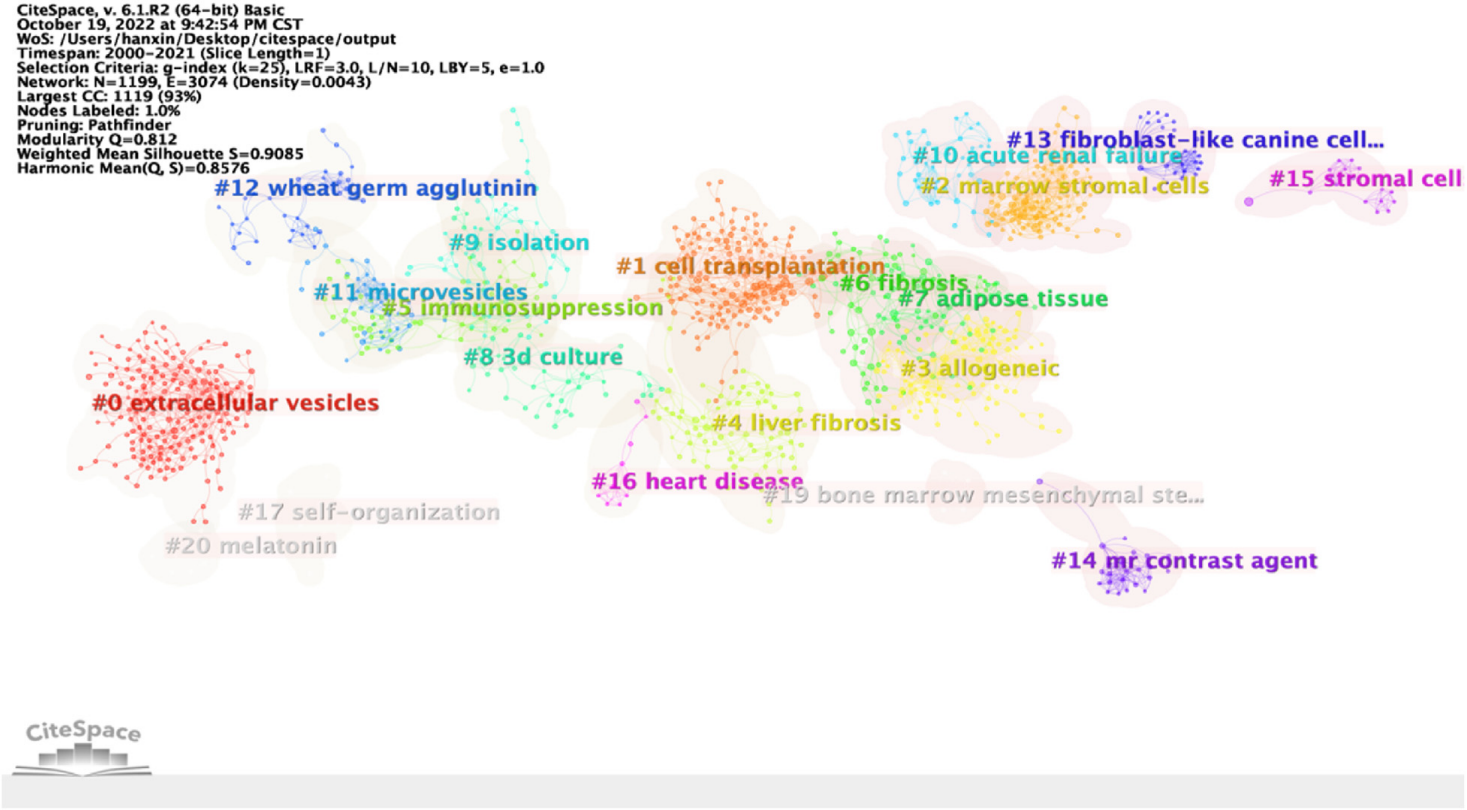
Clustered network of co-cited articles in studies of mesenchymal stem cells in liver disease.

The cluster network comes from 12,852 references (including repeated references) cited by 4452 articles. To detect the research groups and research basis formed in this research field, we analyzed the clustering network and co-occurrence of cited articles describing MSC research in the liver disease field. References are represented by nodes, while discipline sets or specialties are represented by clusters. The visualization of cited articles shows a total of 1199 nodes and 3074 links (Fig. 3), with each cited article represented by a node. The area of each node is proportional to the total citation frequency of the relevant article, and a red circle around the node indicates that it has high emergence. Modularity = 0.812 > 0.3, indicating significant clustering structures. Silhouette = 0.908 > 0.7, meaning the clustering is convincing. Co-cited references could be divided into 20 categories (extracellular vesicles, cell transplantation, allogeneic, liver fibrosis, immunosuppression, fibrosis, adipose tissue, 3D culture, isolation, acute renal failure, microvesicles, wheat germ agglutinin, fibroblast-like canine cell, mr contrast agent, stromal cell, heart disease, self-organization, bone marrow mesenchymal stem cell, and melatonin).

### 3.5 Top-cited articles

The cluster network is based on 12,852 references (including repeated references) cited by 4452 articles. To detect the research groups and research basis formed in this research field, we analyzed the clustering network and co-occurrence of the top 10 cited articles, citation frequency, and citation half-year lifespan (Table 1). Among the top 10 cited articles, seven were published between 2000 and 2010 and three were published after 2010. These articles were published in *Gastroenterology*, *Hepatology*, *Gut*, *Nature*, *Blood*, and *Stem Cells*, where their total citation frequency reached 653 times. T.K. Kuo was cited most frequently in *Gastroenterology* (79), followed by D. Van Poll in *Hepatology* (73) and I. Aurich in *Gut* (70). Sigma is composed of centrality and burstiness and often used to identify innovative literature. Among the top 10 cited articles, “Autologous bone marrow mesenchymal stem cell transplantation in liver failure patients caused by hepatitis B: short-term and long-term outcomes” [18] had the highest Sigma value of 10.1. Sigma values of “Pluripotency of mesenchymal stem cells derived from adult marrow” and “Transplantation with autologous bone marrow-derived mesenchymal stem cells for alcoholic cirrhosis: phase 2 trial” followed, reaching 7.13 and 4.46. The citation half-life is an index to describe the aging degree of literature. The greater the half-life, the greater the effective value of the citation. The half-life of the top 10 cited articles was 2.5 or above. Research areas of the top 10 articles mainly focused on stem cell plasticity, the potential of MSCs to differentiate into hepatocytes, and applications of MSCs in the treatment of liver failure and cirrhosis.

**Table 1:** The top 10 cited articles, citation frequency, and citation half-year lifespan.

Title	Cited frequency	Sigma	Author	Year	Source	Half-life
Stem Cell Therapy for Liver Disease: Parameters Governing the Success of Using Bone Marrow Mesenchymal Stem Cells	79	2.58	Kuo TK	2008	GASTR OENTE ROLOG Y	2.5
Mesenchymal Stem Cell-Derived Molecules Directly Modulate Hepatocellular Death and Regeneration In Vitro and In Vivo	73	1.84	van Poll D	2008	HEPAT OLOGY	2.5
Functional integration of hepatocytes derived from human mesenchymal stem cells into mouse livers	70	1.58	Aurich I	2007	GUT	2.5
Transplantation with Autologous Bone Marrow-Derived Mesenchymal Stem Cells for Alcoholic Cirrhosis: Phase 2 Trial	66	4.46	Suk KT	2016	HEPAT OLOGY	2.5
Autologous Bone Marrow Mesenchymal Stem Cell Transplantation in Liver Failure Patients Caused by Hepatitis B: Short-Term and Long-Term Outcomes	66	10.01	Peng L	2011	HEPAT OLOGY	2.5
Pluripotency of mesenchymal stem cells derived from adult marrow	62	7.13	Jiang YH	2002	NATUR E	2.5
In Vitro Hepatic Differentiation of Human Mesenchymal Stem Cells	61	1.34	Lee KD	2004	HEPAT OLOGY	3.5
Human mesenchymal stem cells xenografted directly to rat liver are differentiated into human hepatocytes without fusion	60	2.47	Sato Y	2005	BLOOD	2.5
Concise Review: Therapeutic Potential of Mesenchymal Stem Cells for the Treatment of Acute Liver Failure and Cirrhosis	58	3.33	Volarevic V	2014	STEM CELLS	3.5
Hepatocyte differentiation of mesenchymal stem cells from human adipose tissue *in vitro* promotes hepatic integration in vivo	58	1.45	Aurich H	2009	GUT	2.5

## 4 Keywords

### 4.1 Co-occurrence and clustering

**Fig. 4:**
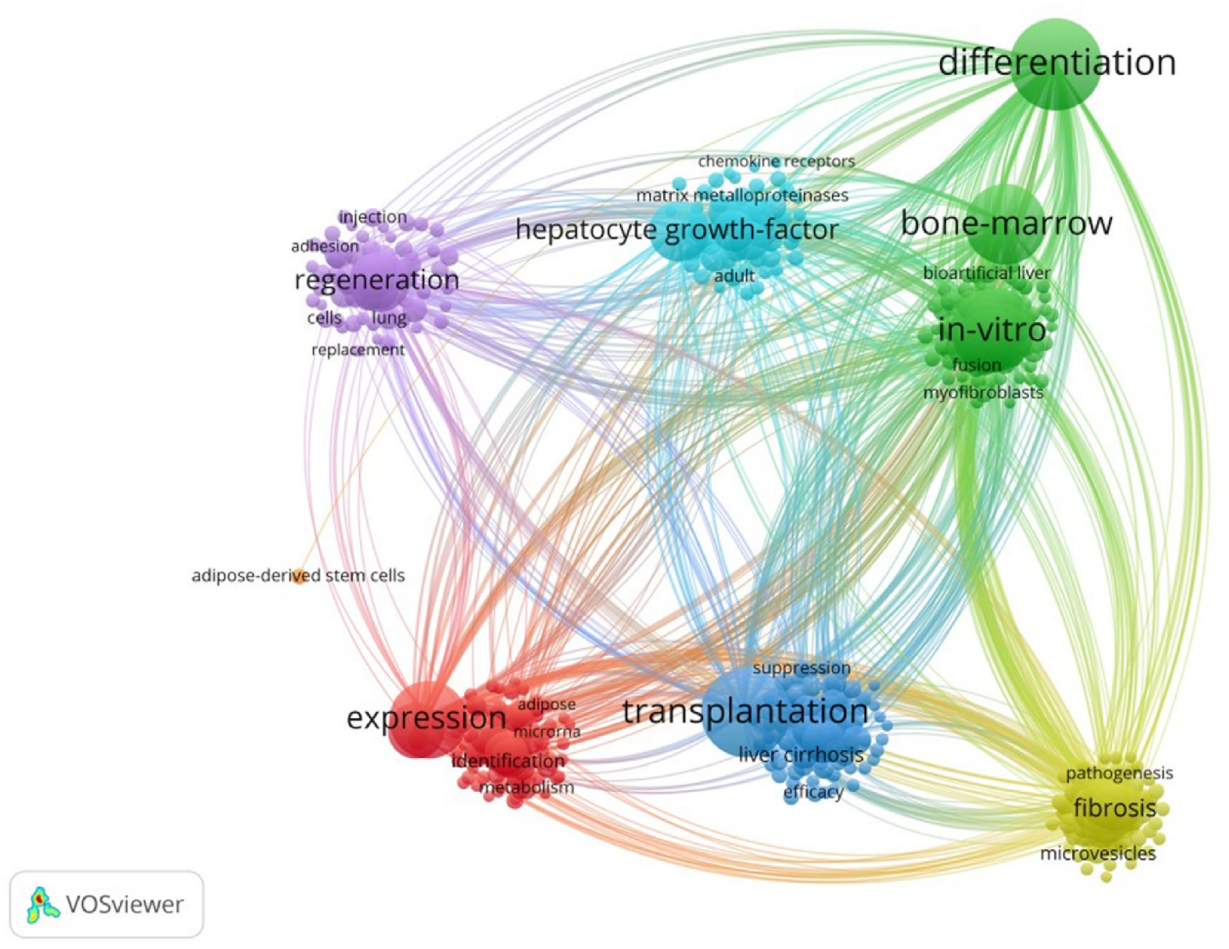
Map of keyword clustering showing 437 keywords with a minimum of 15 occurrence, which were divided into 6 clusters.

We used VOSviewer to conduct co-occurrence and cluster analysis of keywords in 4452 articles. The results identified 437 keywords (each of which appeared at least 15 times) that could be divided into eight categories: hepatocyte growth factor, regeneration, fibrosis, transplantation, differentiation, in vivo, bone marrow, and express (Fig. 4).

### 4.2 Burst detection

**Fig. 5:**
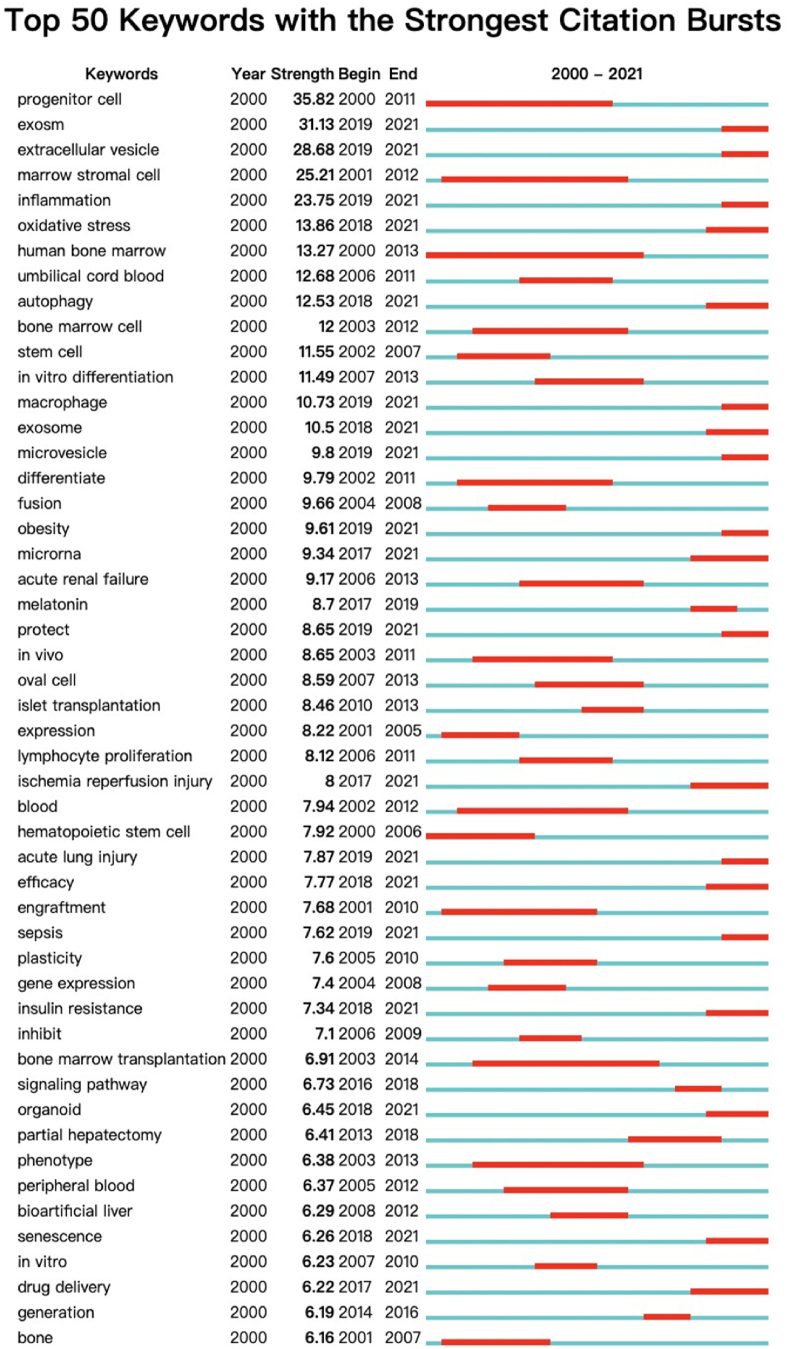
Top 15 keywords with the strongest citation bursts.

Based on keyword burst detection, we discovered an evolutionary trend of MSC research in liver disease and predicted which areas of research will continue to show explosive trends in the future. By analyzing keywords in 4452 articles, we found incidents (excluding search words and nonsense words) between 2000 and 2021. The blue line represents the timeline, and the red line segment represents the time interval of keyword bursts (start year, end year, and duration). As shown in Fig. 5, keywords with high emergence intensity represent important aspects of the research topic and are influential research hotspots. From the perspective of keyword salience and time nodes, from 2000 to 2010, the research in this field mainly focused on the characteristics of MSCs themselves and basic research of MSC applications in transplantation and differentiation. From 2011 to 2021, research on MSCs began to dive into liver cirrhosis, liver cancer, and other specific liver diseases, as well as regenerative medicine, cell therapy, and the role of exosomes. From the perspective of the duration of influence of protruding words, the influence time of gene therapy, progenitor cell, and engraftment is more than 5 years. From the perspective of the position of the time node of protruding words, keywords of time nodes at the end of the time zone included exosomes, extracellular vesicle, autophagy, macrophage, microvesicle, obesity, oxidative stress, microRNA, and ischemia-reperfusion injury. Because of the continuity of burst keywords, we think that research directions pointed to by the aforementioned time nodes at the end of the timeline will continue to become research hotspots in the next few years.

### 4.3 Division and evolution

**Fig. 6:**
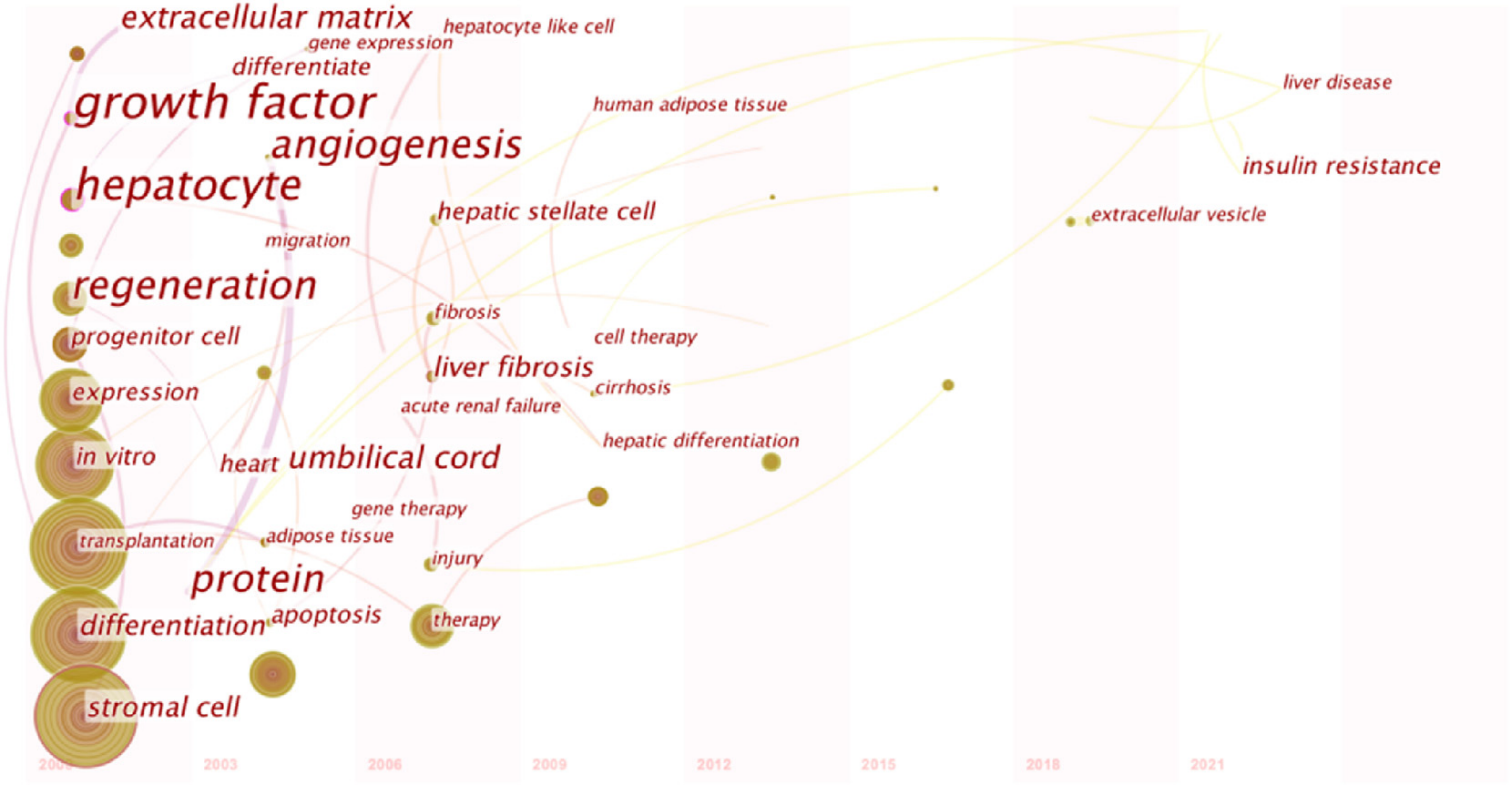
Evolutionary path of studies of mesenchymal stem cells in liver disease.

In Fig. 6, the year corresponding to each keyword is the year in which it first appears in the analysis dataset. Transformation between nodes can show the evolution process of MSCs with a research hotspot. From 2000 to 2010, the study of MSCs in liver disease focused on progenitor cells, differentiation, hepatocyte-like cells, angiogenesis, adipose tissue, liver cirrhosis, and cell therapy. From 2011 to 2015, liver transplantation, oxidative stress, partial hepatectomy, exocrine bodies, and extracellular vesicles received extensive attention in this field. From 2016 to 2021, in addition to exocrine mechanisms and drug delivery receiving continuous attention, miRNA, insulin resistance, ischemia-reperfusion injury, and regenerative medicine have become new foci.

## 5 Discussion

In this work, we performed a bibliometric analysis of the literature on MSCs published in the area of liver disease from 2000 to 2021 using the information visualization tools CiteSpace and VOSViewer. In addition, we utilized Pajek to modify the layout of the cluster map generated by VOS viewer. Based on this analysis of the field of liver disease, we identified the foundational and frontier areas of MSC research. Our primary conclusions are as follows:

(1) Research on MSCs in the field of liver disease is on the rise.

Judging from the observed trends (Fig. 1), the literature on MSCs in the field of liver disease has been increasing for the past 20 years, indicating that research in this field is deepening and more targeted. After 2010, the number of research articles increased significantly. This may be due to certain papers published during 2000-2010 having great influence on further studies of the potential of MSCs to differentiate into hepatocytes, the anti-oxidative stress and paracrine effects of MSCs, rescue of liver failure, and human adipose tissue MSCs. However, after 2010, research on the application of MSCs in liver diseases such as alcoholic liver, liver cirrhosis, and graft-versus-host disease became new hotspots, in addition to the appearance of research describing exosomes, extracellular vesicles, macrophages, and microvesicles.

(2) Research on MSCs in liver disease started in the United States, showing a tendency for cooperation among multiple countries and multiple authors.

China and the United States account for more than half of the total research, indicating their strong interest in this field. Research in Europe and the United States started earlier and is more mature. The co-occurrence analysis of authors from 2000 to 2021 shows that the results published by Li Li, Jin Yang Gu, Daniel R. Meldrum, and Lanjuan Li have made great contributions to the research trends and the current understanding of MSCs in the field of liver disease. These authors have also promoted research cooperation among researchers and between countries.

(3) Studies from 2000 to 2010 laid the foundation for research in this field.

Among the ten most commonly cited articles, Jiang YH et al. found that the study of pluripotent adult progenitor cells solved important problems in the field of stem cell plasticity [19]. In addition, Lee KD et al. characterized the phenotype and multiple differentiation potential of MSCs isolated from human BM and umbilical cord blood, which showed that human MSCs from different sources could differentiate into functional hepatocyte-like cells, challenging the contemporary view that the differentiation potential of adult hepatocytes was limited [20]. Sato Y et al. separated human BM cells into MSCs, CD34 cells, and non-MSCs/CD34 cells and then directly inoculated these cell populations into the liver of rats with chronic liver injury; the results showed that MSCs had a stronger ability to differentiate into hepatocytes than the other two cell populations and no fusion occurred [21]. I. Aurich et al. transplanted MSCs into immunodeficient Pfp/Rag2 mice and found that after they functionally integrated in the mouse liver, they still retained the characteristics of hepatocytes; that is, they continued to store glycogen *in situ* and expressed PCK1, connexin 32, albumin, and the human hepatocyte-specific antigen HepPar1 [22]. Collectively, these results indicate that MSCs have the potential to differentiate into hepatocytes and treat liver diseases. Van Poll D et al. clearly demonstrated for the first time that the systemic infusion of MSC conditioned medium can inhibit the release of liver enzymes, reduce systemic inflammation, inhibit hepatocyte apoptosis, and enhance liver regeneration, thus protecting the liver from acute injury and providing a new treatment for fulminant liver failure [23]. Kuo TK et al. established a mouse model of fatal liver failure induced by CCl_4_ and then transplanted hepatocytes derived from BM-MSCs and MSCs into vein or spleen at different doses, which proved the superiority of vein transplantation in rescuing liver failure [24]. They also proved that BM-MSCs can resist reactive oxygen species and may have a paracrine effect to promote liver regeneration [25]. In addition, it was shown that human adipose tissue MSCs could differentiate into hepatocellular-like cells *in vitro* and promote the integration of hepatocytes in vivo, with a recolonization rate of more than 10% [26]. The authors discussed the immunomodulatory characteristics of MSCs and their potential to differentiate into hepatocytes and collected detailed experimental model data to support the view that MSCs are a promising drug for the treatment of acute liver failure and liver cirrhosis; it is the most frequently cited review article in this field. Suk KT et al. focused on the application of BM-MSCs in the treatment of alcoholic liver cirrhosis. Through a multicenter randomized controlled phase 2 trial of 72 patients with AC who abstained from alcohol for more than 6 months, they proved that autologous BM-MSC transplantation can safely and effectively improve histological fibrosis and liver function in patients with alcoholic liver cirrhosis [27].

(4) Exosomes, obesity, and tissue engineering are research hotspots and frontiers in this field.

In our clustering analysis of reference co-citation and keyword co-occurrence analysis, high-frequency keywords such as regeneration, differentiation, transdifferentiation, and exosomes indicate that liver regeneration and exosomes are current research hotspots for MSCs in liver disease. Keywords were analyzed through emergence detection, as shown in Fig. 5. Progenitor cells, umbilical cord blood, and *in vitro* differentiation also received close attention from researchers in the first 15 years of development in the field, while exosomes, macrophages, oxidative stress, and obesity have become research hotspots in recent years. Research in this field seems to have shifted from basic research to clinical applications, from medical research to multidisciplinary research. Tissue engineering and regenerative medicine are the frontiers of MSC research in liver disease.

The paracrine function of MSCs occurs through the secretion of soluble factors and release of extracellular vesicles. Exosomes are mainly composed of miRNA, mRNA, proteins, and lipids, which can be transferred from secretory cells to target cells [28,29].

Exosomes can carry a variety of biomolecules to target cells and participate in tissue repair and regeneration, which has been a research hotspot in recent years. MSCs and their exosomes were found to promote liver regeneration through the following possible mechanisms: inhibition of hepatocyte apoptosis, regulation of inflammatory responses, regulation of hepatocyte autophagy, improvement of oxidative stress, promotion of neovascularization, and inhibition of liver fibrosis [[30], [31], [32], [33], [34], [35]]. However, the exocrine mechanism of MSCs still requires further study.

Obesity, a group of metabolic syndromes, has become a global health concern. Accordingly, cell transplantation for the treatment of metabolic diseases caused by obesity has become a research hotspot, in which MSCs have become the focus because of their wide availability and rapid proliferation. In addition, primary non-alcoholic steatohepatitis has become a research focus. MSCs play a protective role by affecting insulin sensitivity, which is a potential therapeutic mechanism. In the treatment of islet dysfunction caused by high-fat-diet-induced obesity, Cao et al. found that MSCs could reduce the expression of tumor necrosis factor α and growth factor-like phantom mucin-like hormone receptor, restore expression of liver insulin receptor and peroxisome proliferator-activated receptor gamma, inhibit pancreatic inflammation, and protect islet β cells [36]. Daryabor G et al. found that BM-MSC- and MSC-derived insulin-producing cells can prevent type 1 diabetes by inhibiting the pathological Th1 immune response of insulitis in mice. The regulation of lipid metabolism by MSCs is an important mechanism for the protection of liver function. Liao et al. showed that after MSCs were transplanted into non-alcoholic steatohepatitis rats, serum fatty acid, total cholesterol, and triglycerides were decreased compared with the balanced salt solution treatment group [37]. Studies have also implicated MSCs in the regulation of glucose metabolism. Ding et al. found in a study of MSC treatment of liver failure that MSCs promote glycogen synthesis by inhibiting glycogen synthase kinase 3β, which improved the survival rate of 90% of partial-hepatectomy rats [8]. However, few studies have investigated the mechanisms of key enzymes in glucose metabolism regulated by MSCs, which may become a focus of future research.

At present, the development of tissue engineering is ascendant. With the deepening of tissue engineering research, new progress has been made in using materials to simulate MSC culture microenvironments [38]. Murphy et al. found that by changing the properties of fibrin hydrogel, MSCs can promote the secretion of vascular endothelial growth factor and prostaglandin E2, thus promoting wound healing [39]. Biological three-dimensional printing is an important new technology in tissue engineering. Modular construction of liver tissue engineering technology is a new tissue engineering strategy based on the characteristics of biological three-dimensional printing technology [40]. In 2017, Yanagi et al. first constructed hepatic blast-like cell spheres consisting of hepatic cells, human umbilical vein endothelial cells, and BM-MSCs on a large scale, and then used these spheres as the basic module to print and assemble large-scale liver tissue. The constructed liver tissue had the ability to self-weight *in* vitro and may evolve into the structure of liver tissue. In the past few years, great progress has been made in the study of cell membrane-coated nanoparticles. Liang H et al. designed an MSC/red blood cell-based nanoparticle and demonstrated its intravenous administration reduced inflammation and hepatocyte apoptosis, enhanced liver regeneration and function, and ultimately improved the survival rate of a mouse model of acute liver failure [41].

## 6 Conclusions

There is no doubt that MSCs have great research value in the field of liver disease. Here, we used bibliometrics and information visualization techniques to analyze literature in the Web of Science database. The vicissitude of scientific research achievements, research basis, research hotspots, and frontiers in the past 20 years reveal the multi-country and multi-author cooperation, as well as multidisciplinary research trends in the field of MSCs in liver disease, of which exocrine bodies, obesity, and tissue engineering are the current research hotspots.

## Funding

This work was supported by grants from 10.13039/501100012166National Key Research and Development Program of China (No. 2021YFA1100500), Key Research & Development Plan of Zhejiang Province (No. 2019C03050, No. 2021C03118), the Construction Fund of Key Medical Disciplines of Hangzhou (OO20200093) and The Major Research Plan of the National Natural Science Foundation of China (No. 92159202).

## Author contributions

Chengzuo Han: Writing–original draft, Formal analysis.

Rui Wang: Writing–original draft, Formal analysis.

Nan Xu: Visualization, Investigation.

Xuyong Wei: Supervision, Methodology.

Qiang Wei: Conceptualization, Methodology, Project administration.

Xiao Xu: Conceptualization, Methodology, Resources.

## Data Availability

No additional data are available.
